# Hemolytic anemia due to acute cytomegalovirus infection in an immunocompetent adult: a case report and review of the literature

**DOI:** 10.1186/1752-1947-4-334

**Published:** 2010-10-21

**Authors:** Fabrizio Taglietti, Cecilia M Drapeau, Elisabetta Grilli, Alessandro Capone, Pasquale Noto, Simone Topino, Nicola Petrosillo

**Affiliations:** 1 2ndInfectious Diseases Division, National Institute for Infectious Diseases "L. Spallanzani", Via Portuense, 292-00149 Rome, Italy

## Abstract

**Introduction:**

Cytomegalovirus is a common virus responsible for a wide range of clinical manifestations. Hemolysis is a rare but potentially life-threatening complication of cytomegalovirus infection, described mostly in immunocompromised patients, the pathogenesis of which is still unclear.

We performed a review of the literature regarding cases of hemolytic anemia during acute cytomegalovirus infection in apparently immunocompetent individuals. We searched for relevant articles in PubMed for the period of 1980 through 2008.

**Case presentation:**

We describe a case of Coombs-negative hemolytic anemia in a 44-year-old Caucasian immunocompetent man with acute cytomegalovirus infection.

**Conclusion:**

Clinicians should consider cytomegalovirus infection in the differential diagnosis of hemolytic anemia in immunocompetent adults. Possible therapeutic options include antiviral therapy and steroids, although the best treatment strategy is still controversial.

## Introduction

Cytomegalovirus (CMV) is a common viral agent responsible for a wide range of clinical manifestations that vary according to the immunologic status of the patient. In the immunocompetent adult patient, primary CMV infection is generally asymptomatic or occurs as a mononucleosis-like self-limited syndrome. In immunocompromised patients, CMV infection can lead to severe clinical manifestations related to direct viral cytotoxic effect on specific organs and tissues (gastrointestinal tract, central nervous system, retina, respiratory tract, and hematopoietic system). In patients with transplants, CMV is responsible for allograft rejection and opportunistic infection. Finally, CMV infection has been also associated with other manifestations, including hemolytic anemia [1-3].

Severe hemolysis is a rare but potentially life-threatening complication of CMV infection described mostly in immunocompromised adults [1-3] and children [4]. The pathogenesis of hemolytic anemia during CMV infection is still unclear, although it has been hypothesized to be the result of immunologic activation [1-3]. Hemolytic anemia is rarely described in immunocompetent adults [1-3,5-8].

We describe a Coombs-negative hemolytic anemia in an adult immunocompetent patient with acute CMV infection.

## Case presentation

A 44-year-old Caucasian man, without any relevant past medical history, was admitted to our Infectious Diseases Hospital because of a 30-day history of fever and progressive asthenia. Fifteen days earlier, the patient was hospitalized in an Emergency Medical Department, where acute CMV infection was diagnosed (positive CMV IgM, negative CMV IgG, CMV viremia, 12,698 copies/mL). Other tests showed alanine aminotransferase (ALT), 47 U/L (normal value, < 40); aspartate aminotransferase (AST), 71 U/L (n.v., < 40); alkaline phosphatase (ALP), 304 U/L (n.v., < 130 U/L); and lactate dehydrogenase (LDH), 600 U/L (n.v., < 500 U/L). Mild anemia was present: hemoglobin (Hb), 11.3 g/dL; and increased inflammatory indexes: C-reactive protein, 30.9 mg/L (n.v., < 6), erythrocyte sedimentation rate (ESR), 19 mm/h. No antiviral treatment was started because the patient was immunocompetent.

After hospital discharge, the fever persisted, and the patient complained of progressive asthenia.

At admission to our hospital, the patient appeared pale and asthenic. Physical examination revealed a body temperature of 38°C, heart rate of 100 beats per minute, and moderate hepatosplenomegaly. The blood examinations showed acute hemolytic anemia: red blood cells (RBCs), 2,430,000/mm^3^; Hb, 7.9 g/dL; reticulocyte count, 16.7%; LDH, 778 mU/mL; total bilirubin, 2.4 mg/dL; indirect bilirubin, 2 mg/dL; and undetectable serum haptoglobin.

Noninfectious causes of hemolytic anemia, including hemoglobinopathies (such as glucose-6-phosphate dehydrogenase deficiency), drug toxicity, autoimmune diseases, and malignancies, were excluded. Of note, direct and indirect Coombs tests were negative, although mildly positive for cold agglutinins and cryoglobulins. A total-body computed tomography scan was negative for solid tumors, revealing only moderate hepatosplenomegaly.

Serologic and virologic examinations showed CMV IgM/IgG, positive; CMV antigenemia, negative; CMV viremia, positive (< 400 cp/mL); parvovirus B19 IgM/IgG, positive, with blood polymerase chain reaction (PCR) negative; and EBV VCA IgM/IgG, positive, with blood PCR negative. Blood cultures, antibodies to HIV, hepatitis B and C virus, human herpesvirus-6, herpes simplex virus 1-2, Toxoplasma, Mycoplasma, Legionella, and hepatitis B surface antigen were negative.

The clinical picture was attributed to primary CMV infection. The Hb level was 6.7 g/dL at day four and decreased to 5.4 g/dL at day seven. The patient remained febrile. Considering the rapid decrease of Hb levels, specific antiviral treatment with ganciclovir, 900 mg/day (5 mg/kg/b.i.d., i.v.) was administered to the patient. After hematologic consultation, blood transfusions were prescribed.

The patient remained febrile, with hemoglobin levels ranging between 5 and 6 g/dL.

At day 20, CMV antigenemia, viremia, and blood PCR were negative. Considering the poor clinical response to antiviral treatment, we hypothesized an immunologic pathogenetic mechanism of hemolytic anemia, and steroid therapy with methylprednisolone, 1 mg/kg/day i.v. was started. Gancyclovir therapy was continued.

The clinical condition of the patient improved.

At discharge (day 30), blood examinations showed RBCs, 276,0000/mm^3^; Hb, 9.7 g/dL; reticulocytes, 5.4%; haptoglobin, 105 mg/dL (n.v., 40 to 130). Treatment with 900 mg/qd oral valganciclovir, and oral prednisone, 1 mg/kg/day, was continued.

At day 90, the patient was asymptomatic with an hemoglobin level of 12.2 per deciliter. Valganciclovir and steroids were stopped.

## Discussion

This is an uncommon case of severe hemolytic anemia during primary CMV in an immunocompetent patient. An immunologic mechanism was supported by the clinical improvement with steroid therapy, whereas the clinical picture remained unvaried during antiviral therapy alone. This hypothesis was indirectly confirmed by the demonstration of the abnormal immunologic activation occurring during CMV infection (that is, the positivity of the serologic tests for parvovirus B19 and EBV, together with the negativity of blood PCR for these two viruses), which was likely interpreted as a cross-reaction.

An interesting finding in our case was the negativity of the Coombs test. A positive Coombs test could have helped in identifying an autoimmune mechanism, thus making the patient eligible for early steroid therapy. However, the presence of an underlying autoimmune mechanism could not be ruled out, based only on the negativity of Coombs test. The literature provides evidence of the onset of hemolysis in patients with negative Coombs test during CMV infection [5]. In our specific case, the presence of cold agglutinins may be a possible explanation for the onset of hemolysis.

We performed a review of the literature by PubMed for relevant articles regarding hemolytic anemia during acute CMV infection in apparently immunocompetent individuals, published between 1980 and 2008. Only 12 cases have been reported (Table 1). Rafailidis *et al. *[7] performed a systematic review that included 290 immunocompetent patients with severe clinical manifestations of CMV infection, of whom only five were found to have hemolytic anemia.

**Table 1:** Hemolytic anemia during acute cytomegalovirus infection in adult immunocompetent patients: data from the literature

Authors/Year	Number of patients	Coombs test	BT	Steroids	Anti-CMV therapy	Outcome
Rafailidis M./2008 [7]	5*	NS	NS	NS	NS	Cured

Veldhuis *et al.*/2004 [5]	1	Neg	No	No	No	Cured

Salloum *et al.*/1994 [9]	2	Case 1: Pos Case 2: Pos	No No	Yes No	No	Cured

Van Spronsen D. *et al*./1996 [2]	1	Neg	No	Yes	Yes	Cured

Gavazzi G. *et al.*/1999 [1]	1	Pos	No	Yes	Yes	Cured

Horwitz *et al.*/1984 [3]	2	Case 1: Pos Case 2: Neg	Yes No	Yes No	No	Cured

BT, blood transfusion; Neg, negative; NS, not specified.

*Among 290 apparently immunocompetent patients with severe cytomegalovirus infection who were included in a review.

Among the 12 cases reported in the literature, the Coombs test was positive in four, negative in three, and was not specified for the remaining five patients (Table 1).

Bonnet *et al. *[8] described 115 patients with acute CMV infection. Twenty-three patients (20%) had hemolytic anemia; however, the authors did not distinguish whether the hemolysis was secondary to hypersplenia or directly connected to the CMV infection; thus this study was not reported in Table 1.

Regarding therapeutic management, two patients were treated with steroids and anti-CMV therapy (one of whom also had blood transfusions); two received only steroid therapy; three patients were not given any specific treatment; and for the remaining five patients, the treatment was not specified. Interestingly, the prognosis was favorable in all cases, including those patients who did not receive steroids and/or antiviral therapy. One of those cases had a clinical history similar to that of our patient [5], with a hemoglobin level that reached 5.1 g/dL, and the patient experienced a full and spontaneous recovery without additional medications.

As is evident in the literature, no conclusive statements regarding specific treatment of hemolytic anemia during acute CMV infection in immunocompetent patients can be made.

In our opinion, although steroid and specific antiviral therapy was given in our patient, the policy of "wait and see" in the presence of hemolytic anemia without severe manifestations during CMV infection in an immunocompetent patient could be justified.

## Conclusions

Clinicians should consider CMV infection in the differential diagnosis of hemolytic anemia in immunocompetent adults. The true incidence of this complication may be underestimated, because CMV serology may not be routinely obtained in patients with hemolysis. Possible therapeutic options include antiviral therapy and steroids, although the best treatment strategy is still controversial.

Randomized controlled trials are needed for conclusive answers regarding the specific treatment of hemolytic anemia due to CMV infection.

## Competing interests

The authors declare that they have no competing interests.

## Consent

Written informed consent was obtained from the patient for the publication of this case report and accompanying images. A copy of the written consent is available for review by the Editor-in-Chief of this journal.

## Authors' contributions

FT followed up the patient during the hospitalization, analyzed data from the literature, and wrote the article. ST and PN analyzed data from the literature. CMD was the major contributor in writing the manuscript. AC and EG followed up the patient after the discharge from the hospital. NP reviewed the manuscript. All authors read and approved the final manuscript.
